# Prescription Surveillance and Polymerase Chain Reaction Testing to Identify Pathogens during Outbreaks of Infection

**DOI:** 10.1155/2013/746053

**Published:** 2013-02-07

**Authors:** Hiroaki Sugiura, Tsuguto Fujimoto, Tamie Sugawara, Nozomu Hanaoka, Masami Konagaya, Kiyoshi Kikuchi, Eisuke Hanada, Nobuhiko Okabe, Yasushi Ohkusa

**Affiliations:** ^1^Sugiura Clinic, 2-8-3 Imaichi-Kita, Honmachi, Shimane, Izumo 693-0002, Japan; ^2^Infectious Disease Surveillance Center, National Institute of Infectious Diseases, Shinjuku, Tokyo 162-8640, Japan; ^3^Shimane Prefectural Central Hospital, Shimane, Izumo 693-8555, Japan; ^4^Shimane University Hospital, Shimane, Izumo 693-8501, Japan; ^5^Kawasaki City Health Institute of Public Health, Kanagawa, Kawasaki 210-0834, Japan

## Abstract

Syndromic surveillance, including prescription surveillance, offers a rapid method for the early detection of agents of bioterrorism and emerging infectious diseases. However, it has the disadvantage of not considering definitive diagnoses. Here, we attempted to definitively diagnose pathogens using polymerase chain reaction (PCR) immediately after the prescription surveillance system detected an outbreak. Specimens were collected from 50 patients with respiratory infections. PCR was used to identify the pathogens, which included 14 types of common respiratory viruses and *Mycoplasma pneumoniae*. Infectious agents including *M. pneumoniae*, respiratory syncytial virus (RSV), rhinovirus, enterovirus, and parainfluenza virus were detected in 54% of patients. For the rapid RSV diagnosis kit, sensitivity was 80% and specificity was 85%. For the rapid adenovirus diagnosis kit, no positive results were obtained; therefore, sensitivity could not be calculated and specificity was 100%. Many patients were found to be treated for upper respiratory tract infections without the diagnosis of a specific pathogen. In Japan, an outbreak of *M. pneumoniae* infection began in 2011, and our results suggested that this outbreak may have included false-positive cases. By combining syndromic surveillance and PCR, we were able to rapidly and accurately identify causative pathogens during a recent respiratory infection outbreak.

## 1. Introduction

Japanese traditional surveillance is based on definitive diagnosis and is enforced by the infection control laws in Japan for the early detection of agents of bioterrorism and outbreaks of emerging infectious diseases. After the infectious disease is diagnosed at sentinel medical institutions, at least 10 days are required until it is announced nationwide. Therefore, a major fault of this surveillance system is the delay in disseminating information.

A surveillance system that can identify the early stages of an outbreak of infectious disease is necessary. Therefore, syndromic surveillance systems have been implemented in many countries since 1995 [1]. Syndromic surveillance monitors changes in the number of patients according to symptoms such as fever, vomiting, diarrhea, and rash for further investigations. Information regarding the identification of local infectious disease outbreaks, such as school absenteeism, emergency room visits, and prescriptions of therapeutic drug against infectious diseases, are also subjects of the survey [2]. Syndromic surveillance can offer a rapid method to detect an outbreak of infection compared with traditional surveillance; such surveillance systems are currently used worldwide [3, 4].

In some cases, an infectious outbreak can be detected on the same or the following day. Although syndromic surveillance provides rapid results, it has the disadvantage that definitive diagnoses are not considered. In other words, in general, its specificity may be lower than that of traditional surveillance systems. Laboratory testing performed on all symptomatic patients can yield a very high specificity, but is cost prohibitive, whereas laboratory testing on selected patients for syndromic surveillance can detect some specific aberrations at a lower cost, thereby overcoming the shortcomings of both systems. The current study highlights an example to further analyze this possibility.

**Figure 1:**
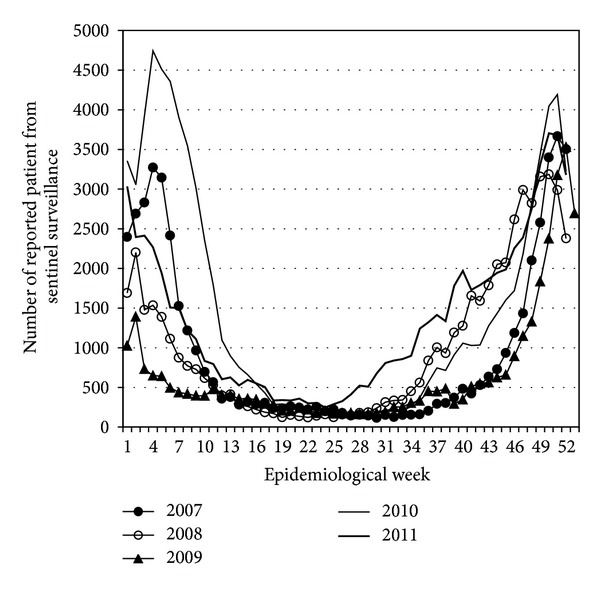
Number of reported RSV cases from sentinel medical institutions in Japan. There are approximately 500 sentinel medical institutions in Japan, which are selected from those equipped with departments of pediatrics and internal medicine and with more than 300 beds.

**Figure 2:**
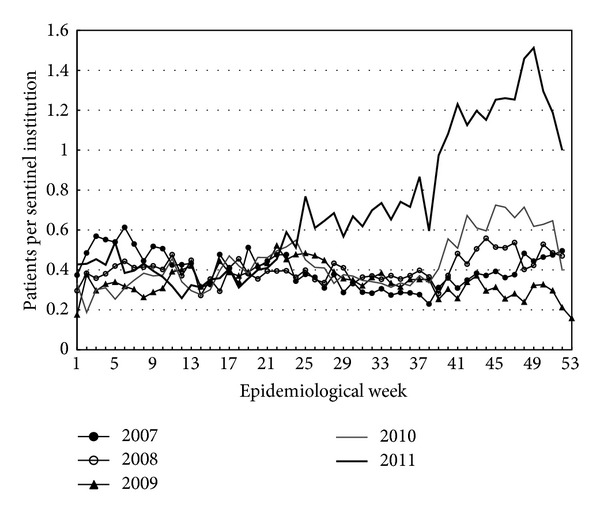
Patients per sentinel medical institution reporting *M. pneumoniae* infections.

In the fall of 2011, the number of patients with symptoms of upper respiratory tract infections markedly increased in Japan. Infectious disease weekly reports (IDWRs) (http://www.nih.go.jp/niid/ja/idwr.html in Japanese), which constitute the traditional and official Japanese sentinel surveillance system, reported a higher incidence of respiratory syncytial virus (RSV) (Figure 1) and *M. pneumoniae* infections (Figure 2). A primary feature of *M. pneumoniae* respiratory infections is the degree of the symptom worsening from mild upper respiratory tract inflammation to pneumonia. *M. pneumoniae* infection is associated with exanthem, hemolytic anemia, gastrointestinal damage, arthritis, and various neurological symptoms [5].

Outbreaks of *M. pneumoniae* persisted throughout June 2012, although it is unclear why this organism has continued to be responsible for such a widespread national outbreak in Japan since the fall of 2011 [6]. Koike et al. [7] detected only 40 patients (14.5%) among 275 suspected cases of *M. pneumoniae* infection from 2006 to 2008 in Japan. A clinical diagnosis of *M. pneumoniae* infection is difficult without laboratory confirmation. In many sentinel hospitals, the *M. pneumoniae*-specific IgM antibody rapid detection test is used, but during screening, a positive result in the test does not always indicate acute infection by this organism. We suspect that the outbreak of *M. pneumoniae* infection included false-positive cases [7, 8].

IDWRs are very important for clinicians, enabling them to identify the seasonal prevalence of known diseases. However, these reports become available after a minimum of 10 days following patient examinations. Therefore, traditional and official surveillance systems have the distinct disadvantage of being slow and are limited to reporting pathogens chosen in advance.

**Figure 3:**
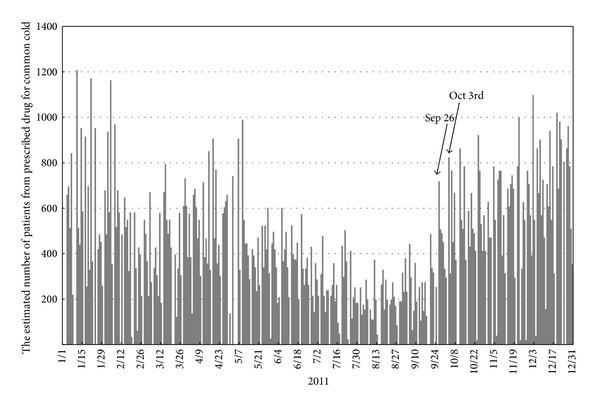
Combination cold medication prescriptions recorded by the prescription surveillance system over time. On September 26, 2011, we noticed an unusual peak and then carefully monitored the real-time prescription surveillance and found a second peak on October 3, 2011. We confirmed this abnormality and began this study on the following day (October 4, 2011).

In the fall of 2011, by monitoring the increase in the number of combination cold medications (active ingredients: salicylamide, acetaminophen, anhydrous caffeine, and promethazine methylene disalicylate) prescribed since 2009, the prescription surveillance system detected an increase in the number of patients with symptoms of upper respiratory tract infections. On September 26, 2011, we noticed the first unusual peak and began to carefully monitor the real-time prescription surveillance system and observed a second peak on October 3, 2011 (Figure 3). However, monitoring prescriptions for combination cold medications does not lead to the identification of the pathogens responsible for the illnesses being treated. Thus, we conducted pathogen identification using the PCR method after being alerted by the prescription surveillance system on October 4, 2011 (the following day).

The purpose of the present study was to evaluate whether the PCR method triggered by the results of the prescription surveillance system can rapidly and accurately identify causative pathogens of local outbreaks of infection. Our results allowed for earlier diagnoses at medical facilities and the dissemination of this information among other institutions to avoid inappropriate use of antibiotics and instigate measures against the spread of infectious diseases.

## 2. Materials and Methods

### 2.1. Prescription Surveillance

Although very common in the US and other countries, there is no nationwide syndromic surveillance system to electronically monitor medical records in Japan. Because of the low prevalence of electronic medical records and a restrictive privacy policy, we perform prescription surveillance nationwide for syndromic surveillance by monitoring the number of prescriptions for certain types of drugs such as anti-influenza medications.

There are approximately 45,000 pharmacies that deliver almost half of the prescribed drugs nationwide and almost all record prescriptions electronically. The prescription surveillance system was developed by the Infectious Disease Surveillance Center of the National Institute of Infectious Diseases in collaboration with EM Systems Co. Ltd. (Osaka, Japan), a leading provider of prescription surveillance used by pharmacies through the Application Server Provider (ASP) system. The ASP system is very useful for syndromic surveillance because data transfer is unnecessary. Thus, it can dramatically decrease costs and maintain a high level of confidentially. Its widespread use started in April 2009, and approximately 6,300 (13%) Japanese pharmacies actively participated in the program as of October 2011.

The ASP system tracks prescription information, but patient symptoms and diagnoses are not recorded. Categories of syndromic surveillance include the type of prescribed drugs. Currently, the syndromic surveillance system monitors several types of drugs, including those for relief of fever and pain due to common colds, as well as antiviral agents, anti-influenza medications (except amantadine), and anti-varicella zoster virus (VZV) drugs. The surveillance of the last two is also classified by age: <15, 16–64, and >65 years. Data collection and analysis are automatically performed every night, and the results are available on the home page of a secure internet site early the next morning.

Monitoring the usage of anti-influenza and anti-VZV drugs is particularly useful for early detection of outbreaks of infection because these drugs are used only to treat specific viral infections.

### 2.2. Clinical Samples

Between October 4 and 28, 2011, 50 patients were included in the present study who either presented at a single clinic with a chief complaint of respiratory symptoms or fever or were suspected of having respiratory tract infections after being identified through the syndromic prescription surveillance system. In Japan, a rapid diagnosis kit suitable for use at outpatient clinics is currently available, and the costs are covered by the national health insurance program. The tests allow for rapid detection of infections caused by the influenza virus, RSV, and adenovirus. A total of 18 pharyngeal swabs to screen for adenovirus infections and 32 nasal swabs to screen for RSV and influenza viral infections (rapid RSV) were collected [9]. Viruses were extracted from the swabs using immunochromatography (IC) kits with approximately 500 *μ*L of a mucolytic agent provided by the manufacturer. After the assay, approximately 200 *μ*L of the agent remained in the IC-kit tubes. This medical waste was transferred to universal transport medium (359C; Copan Italia S.p.A, Brescia, Italy) and analyzed using real-time polymerase chain reaction (PCR) [10] and Hyper-PCR [11], which is a faster technique compared with the previously available PCR applications. Thus, we used Hyper-PCR for the applicable pathogens. The CycleavePCR respiratory infection-pathogenic virus detection kit (Takara Bio, Shiga, Japan) was used to detect 11 types of viruses: human RSV types A and B, human parainfluenza virus types 1–3, human metapneumovirus, influenza A and B viruses, human adenovirus, human bocavirus, and human rhinovirus. The Thermal Cycler Dice Real Time System II MRQ (Takara Bio) was used to detect and identify the 11 types of viruses detected by the CycleavePCR kit [10]. Hyper-PCR [11] was performed using the One Step SYBR High Speed RT-PCR Kit (Hyper-PCR) (Takara) to detect RSV types A, B, human parainfluenza virus types 1, 3, human rhinovirus, enterovirus, and influenza A (H1N1) 2009 (primers are listed in Table 1) using the Hyper-PCR MK IV PCR system (Trust Medical, Hyogo, Japan). The accuracy of the Hyper-PCR methods was confirmed by comparison with other conventional PCR methods. Conventional PCR was used to detect *M. pneumoniae* [12]. In addition, the presence of coronavirus infection was tested in patients from whom no infectious agents were detected [13].

## 3. Ethical Considerations

This study only collected anonymous information that cannot be associated with individual patients. Patient samples were collected during the course of medical care provided at the participating facilities, and all examinations and testing for pathogens occurred at the request of the medical facilities for the purposes of diagnosis and treatment. This study used only existing medical records and documents, and oral informed consent was obtained from all patients.

## 4. Results

After testing the specimens, we provided the results to a medical institution within 4 days including the conveyance period. The 50 patients tested in this study included 2 infants (1 male and 1 female, aged <1 year), 25 children (12 males and 13 females, aged 1–6 years), 10 elementary school pupils (6 males and 4 females, aged 7–12 years), 4 minors (2 males and 2 females, aged 13–18 years), 8 adults (3 males and 5 females, aged >18 years), and 1 patient (age unavailable).

Table 2 lists the pathogens detected by the PCR analysis stratified by age in the 27 patients. In children, enterovirus, rhinoviruses, RSV, and parainfluenza viruses were detected, whereas *M. pneumoniae* was detected only in elementary school pupils and minors. In the remaining 23 patients, no pathogens were detected. These 23 patients were also found to be negative for coronavirus.

PCR was used to obtain definitive viral diagnoses via rapid RSV and adenovirus diagnosis kits, and the sensitivity and specificity were calculated for these test kits. For the rapid RSV diagnosis kit, sensitivity was 80% and specificity was 85%. For the rapid adenovirus diagnosis kit, no positive results were obtained; therefore, sensitivity could not be calculated and specificity was 100%.

RSV infections were detected using the rapid diagnosis kit, but rhinovirus, enterovirus, and parainfluenza virus infections were not. The causative pathogens were unknown in many patients, although they were nevertheless treated for upper respiratory tract infections.

Evaluation of the incidence of various symptoms in patients infected with different pathogens showed that rhinoviruses were detected in nasal swab specimens more often than other viruses and patients with rhinovirus infections were less likely to present with fever (Table 3).

All RSV-positive patients were children, 80% of whom presented with coughing. All patients who were tested using the rapid adenovirus detection kit showed negative results. However, all these patients also tested negative for adenovirus using sensitive PCR tests. Thus, adenovirus was not considered to be the causative organism of this suspected outbreak.

## 5. Discussion

Here, we examined a combination of syndromic surveillance and PCR testing and showed the potential to identify pathogens during the early stage of an outbreak of respiratory infections. In the future, it would be desirable to develop an *M. pneumoniae* diagnosis kit that can diagnose pathogens from nasal or pharyngeal swabs at outpatient clinics or the bedside of patients.

In Japan, two official pathogen surveillance methods have been conducted under the infection control laws: sentinel pathogen surveillance and active surveillance. The official pathogenic surveillance has been conducted at sentinel medical institutions regardless of outbreaks. On the other hand, in patients with serious diseases, active pathogenic surveillance has sometimes been conducted on the basis of notifications by medical institutions. However, active surveillance is conducted only when an infection spreads widely enough to cause serious problems in a particular region and the surveillance of pathogens may not be timely enough to mount a response to control outbreaks. Pathogenic surveillance for all patients with signs of an infection would detect agents of bioterrorism and emerging infectious diseases; however, the cost would be prohibitive. Therefore, system coordination to perform pathogen surveillance based on early detection of outbreaks is necessary. The scheme proposed by the present study uses PCR testing triggered by detection alerts from syndromic surveillance systems. In general, syndromic surveillance offers earlier detection of infectious diseases than traditional surveillance. Moreover, if the pathogen remains unknown following bedside testing using several rapid tests or other typical examinations, the proposed scheme requires the collection of specimens as soon as possible and sending them to a laboratory for definitive diagnoses. However, it takes a few days to transfer the specimens and a few extra days for the information of the identified pathogen to be shared among medical facilities, public health centers, and local governments in the involved areas. In the proposed scheme, we can use pathogenic information to control ongoing outbreaks and, hopefully, decrease the number of potential infections.

Thus far, syndromic surveillance with pathogenic testing has been conducted by collecting samples from patients receiving telephone consultations [18] and those receiving emergency department consultations [19]. Syndromic surveillance using electronic medical records has been combined with testing for the influenza virus [20]. However, these systems have focused only on rapid testing and are mainly used for influenza monitoring [20, 21]. Therefore, syndromic surveillance trials for nonspecific pathogens using PCR for undiagnosed infectious diseases, similar to the present study, have not been performed before.

In the present study, an outbreak was detected by routine syndromic surveillance, in which samples were regionally collected for PCR analysis. These tests for viral infections allowed for differentiation between bacterial and viral infections, thus facilitating treatment without the unnecessary use of antibiotics. Although the present laboratory tests cannot be performed for all individual clinical diagnoses, the results were immediately made available to clinicians for the treatment of other patients with similar symptoms.

The symptoms reported in the present study were rather mild; therefore, no patient required hospitalization, and no further testing was performed in undiagnosed patients. However, if severe cases were to occur, careful identification of pathogens would be desirable. Rhinoviruses were detected in nasal swab specimens more frequently than other viruses. Therefore, it is likely that children who present with nasal discharge and mild fever may be reservoirs for rhinoviruses [22]. Testing for respiratory viral infections in emergency room outpatients by PCR analysis showed that the most frequently detected viruses were picornaviruses, including rhinoviruses [23]. When children present with coughing as the main symptom, RSV should be considered as the most likely pathogen. 

The finding that *M. pneumoniae* infection was not detected in infants and children, but rather in elementary school pupils and minors, was consistent with reports that *M. pneumoniae* may often cause asymptomatic infections before the age of 5 years, after which immunity decreases as children become susceptible to symptomatic *M. pneumoniae* infections [5]. 

In Japan, nationwide outbreaks of *M. pneumoniae* began in 2011 and continued as of January 2012, during which time *M. pneumonia*, rhinovirus, enterovirus, parainfluenza virus, and RSV have been identified. Our results suggested that this outbreak may include false-positive cases and subsequent inappropriate prescriptions of antibiotics. 

An increased frequency of macrolide-resistant *M. pneumoniae* became widely reported in the Japanese media in the fall of 2011 [24]. Therefore, this news may have induced an abnormal increase in the number of patients (Figure 2). The rapid test available in Japan for *M. pneumoniae* uses sera samples [25]. Although general clinics may outsource *M. pneumoniae* antibody testing and cold hemagglutinin testing, blood testing is usually not performed in cases of mild pediatric illnesses. 

In the future, it would be desirable to develop *M. pneumoniae* diagnostic kits using nasal or pharyngeal swabs at outpatient clinics or bedside. Until such kits for the diagnoses of *M. pneumoniae* and other infectious diseases are developed, syndromic surveillance with PCR testing offers a useful countermeasure against infectious outbreaks. In this study, we could not detect single infectious agents that explained the outbreaks; however, our results excluded *M. pneumoniae*.

The present study was limited to a single clinic. Therefore, further studies involving more facilities should be undertaken. It is also necessary to develop a network and sample transportation system among the facilities partaking in the syndromic surveillance system and to adequately staff laboratories with experienced technicians. 

Syndromic surveillance data has been mathematically or statistically analyzed in many studies. However, when an abnormal value is reported by syndromic surveillance, there are many cases in which the pathogens cannot be identified by the calculations introduced in these articles.

## 6. Conclusion

When *M. pneumoniae* and RSV infections were prevalent nationwide during the fall of 2011, we observed an abnormal increase in common cold prescriptions through the Japanese surveillance system and were able to evaluate the incidence of various pathogens via PCR testing.

## Figures and Tables

**Table 1:** Hyper-PCR primers.

Pathogen	Primer	Base sequence (5′-3′)	Polarity	Reference
RSV-A	RSA-F	TGC AAG CAG AAA TGG AAC AAG T	+	[14]
106 bp	RSA-R	AAT AAT GAT GCT TTT GGG TTG TTC A	−	

RSV-B	RSB-F	GATGGCTCTTAGCAAAGTCAAGTTAA	+	[15]
104 bp	RSB-R	TGTCAATATTATCTCCTGTACTACGTTGAA	−	

Parainfluenza 1	PIS1+	CCGGTAATTTCTCATACCTATG	+	[16]
317 bp	PIS1−	CCTTGGAGCGGAGTTGTTAAG	−	

Parainfluenza 3	Para3.1	CTCGAGGTTGTCAGGATATAG	+	[16]
189 bp	Para3.2	CTTTGGGAGTTGAACACAGTT	−	

Rhinovirus	SRHI-1-NIID	CGGGTAGCTTCCACCACCAGCCCTT	+	[16]
549 bp	SRHI-2	GGGACCAACTACTTTGGGTGTCCGTGT	−	

Enterovirus	entR1	ATTGTCACCATAAGCAGCCA	+	[17]
172 bp	entE2	CCTCCGGCCCCTGAATG	−	

H1N1 2009	swH1-F2	TCATGCGAACAATTCAACA	+	Present study
127 bp	swH1-R2	TGGGGCTACCCCTCTTAGTTTG	−	

**Table 2:** Numbers of pathogens detected by PCR according to age.

	Infants	Children	Elementary school pupil	Minor (junior high school student or older)	Adult
Enterovirus	0	2	1	0	1
*Mycoplasma pneumoniae *	0	0	1	1	0
Parainfluenza 1	0	2	0	0	1
Rhinovirus	2	9	0	0	1
Rhinovirus + parainfluenza 1	0	1	0	0	0
Rhinovirus + RSV-A	0	1	0	0	0
Rhinovirus + RSV-A and RSV-B	0	1	0	0	0
RSV-A	0	2	0	0	0
RSV-B	0	1	0	0	0

**Table 3:** Incidences of symptoms detected in infections according to individual pathogens (*n* = 50).

	Number of infections	Fever	Headache	Nasal discharge	Pharyngeal pain	Cough
*Mycoplasma pneumoniae *	2	0%	0%	0%	50%	100%
Enterovirus	4	67%	25%	75%	25%	25%
Parainfluenza 1	3	33%	33%	33%	0%	67%
Rhinovirus + parainfluenza 1	1	0%	0%	0%	0%	100%
Rhinovirus	12	20%	0%	67%	0%	83%
Rhinovirus + RSV-A	1	0%	0%	100%	0%	100%
Rhinovirus + RSV-A + RSV-B	1	100%	0%	100%	0%	100%
RSV-A	2	100%	0%	100%	0%	100%
RSV-B	1	100%	0%	0%	0%	0%
None	23	21%	13%	57%	30%	35%
